# Supplementary material for: Identification of photoperiod-induced specific miRNAs in the adrenal glands of Sunite sheep (Ovis aries)
Source: Front Vet Sci. 2022 Jul 22;9:888207. doi: 10.3389/fvets.2022.888207 (PMC9354845; doi:10.3389/fvets.2022.888207)
Supplement: Supplementary file 1 [file Data_Sheet_1.ZIP › Supplementary file/miRDeep2 file.zip!/miRDeep2 file/Novel_Structure/SPLP42A3/Novel_332.pdf]

Provisional ID	:	NC_019458.2_571
Score total	:	1.5
Score for star read(s)	:	-1.3
Score for read counts	:	0
Score for mfe	:	1.2
Score for randfold	:	1.6
Score for cons. seed	:	
Total read count	:	817
Mature read count	:	817
Loop read count	:	0
Star read count	:	0

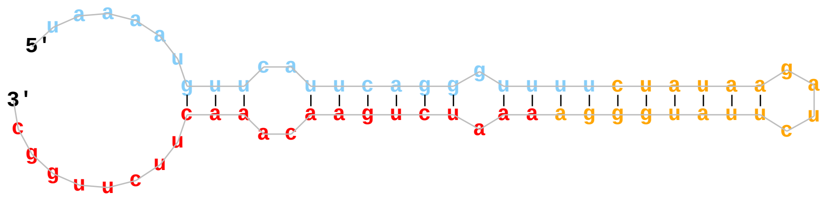

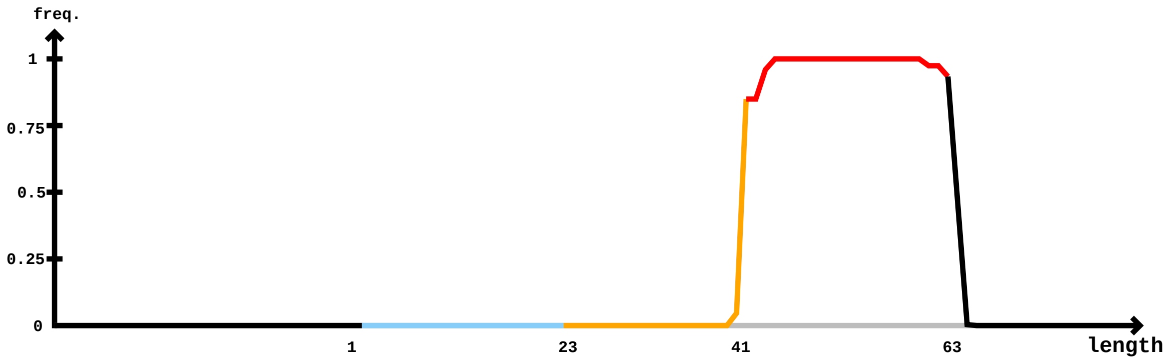

Star **Mature**

```
5'- uuucuguaaguucaauauuauuggguaggcuaaaauguucauucaggguuuucuauaagaucuuaugggaaaaucugaacaaacuucuuggccaacccaauccuuccaaau   -3'    exp
    ..................(((((((.((((((...(((..((((((.((((((((((....)))))))))).))))))..)))...)))))).)))))))...........   reads    mm    sample
    .....................................................................aaaaucugaacaaacuuUuu......................   18       1     seq
    .....................................................................aaaaucugaacaaacuuUuugg....................   19       1     seq
    .....................................................................aaaaucugaacaaacuucuuggc...................   1        0     seq
    ......................................................................aaaucugaacaaacuuUuu......................   3        1     seq
    ......................................................................aaaucugaacaaacuuUuugg....................   14       1     seq
    ......................................................................aaaucugaacaaacuuUuuggc...................   637      1     seq
    ......................................................................aaaucugaacaaacuucuuggc...................   2        0     seq
    ........................................................................aucugaacaaacuuUuuggc...................   16       1     seq
    ........................................................................aucugaacaaacuucuuggc...................   1        0     seq
    ........................................................................aucugaacaaacuuUuuggcc..................   73       1     seq
    .........................................................................ucugaacaaacuuUuuggcc..................   30       1     seq
    .........................................................................ucugaacaaacuuUuuggcca.................   3        1     seq
```
